# Notes and News

**Published:** 1893-12-16

**Authors:** 


### Notes and News.

Collected and Communicated.

The annual meeting of the constituents of the Hospital Sunday Fund will be held at the Mansion House on Tuesday, 19th inst., at 3 p.m., when all who are interested are invited to be present to support the Lord Mayor.

Last Saturday H.R.H. the Duchess of Albany paid a visit to the new White Star Line mail steamer "Gothic," thus showing her kindly interest in the Seamen's Hospital Society, for whose benefit this splendid vessel will be on exhibition on Saturday next, December 16th.

The Hospital Saturday Fund has been enriched to the extent of upwards of £376, being the proceeds of athletic meetings held on its behalf at Herne Hill and Paddington during the year. The major part of the sum was gathered at Herne Hill, and it has been decided to hold one large meeting in 1894, which is to include both cycling and athletics. It is most unsatisfactory that no balance-sheet has been published, and that the Hospital Saturday Fund authorities should refuse information as to the expenses, which are said to amount to no less than £400. The Pneumatic Saddle Company have offered a hundred guinea Challenge Shield for competition, which, with the trophies already existing, will furnish good inducements to competitors, and render the meeting of material assistance to the Fund.

We are glad to note that some improvement has been made in some shops by way of providing seats for those serving in them. This we learn through Miss Clara Collet's reports to the Labour Commission. Yet whilst regarding the step forward as encouraging, we still feel that were it not for the apathy of the women of the upper classes and those who are purchasers to a sufficiently large extent to be influential, the tyranny exercised by shopkeepers would not be so general. An organised appeal to the larger establishments to begin with, to be followed by the more coercive measure of a boycott if the appeal were ignored, would very soon bring about the desired result, and the very trying lot of the shop-girl would be much ameliorated.

The Local Government Board have refused their consent to the erection of the proposed Scarlet Fever Convalescent Home on the Grangewood Estate, Upper Norwood.

The extreme importance of correct diagnosis in the case of patients admitted to the fever hospitals is more strongly impressed upon us day by day. Doubtful cases should certainly be kept under strict observation. At Wimbledon small-pox has been spread amongst scarlet fever and diphtheric patients by the admission of a small-pox case into the hospital, and now especial accommodation has had to be arranged for quite a number of small-pox patients; and, at Leicester, in spite of the experience at Wimbledon, tents have been ereeted for small-pox patients on the same site as the fever hospital. It is a terrible prospect when sufferers conveyed to fever hospitals must feel they are running the risks of having their ills added to in process of treatment.

The desirability of a Hydropathic Hospital for London has been mooted more than once. If the believers in the "water cure" are not in the majority, at least it must be admitted that the attendant treatment of those who resort to such hydropathic establishments is practically conducive to health. There are numbers of persons who could be perfectly well restored to health could they be induced to submit for a time to a common-sense *régime*. The medical man frequently realizes his incompetence to effect a cure through his counsels being disregarded so soon as the door closes behind him. In such cases a "Hydro" is an excellent institution, and would act as a powerful auxiliary in the hands of the physician, especially in the case of the bread-winner of small means, and many a young man in our cities whose system is impoverished for want of proper nourishment and better air. Hydropathic establishments as they exist, supported by the better classes, are far too expensive to assist the class they could above all benefit, and so if such an institution is to be seriously contemplated, it must be brought within the reach of those whose salaries do not exceed £1 per week, and then it will be no less beneficial as a health agent if it passes under the name of a hospital.

Improved opportunities for health are not always without disadvantages. This is specially the case now that the tops of omnibuses are frequented by ladies, who formerly had no alternative but to make the best of the less pleasant interior of the vehicle. The chair seats are far more agreeable than the long back-to-back benches, and no one appreciates them more than the pickpocket. The opportunities thus offered are increasingly used, and so many cases of lost purses have come to our notice lately in connection with the much-patronised omnibus that we think a timely warning may be useful. Whatever dangers the habit may afford in the case of foot passengers, it is certainly better to retain the purse in the hand whilst travelling by omnibus. It is not enough to be on one's guard when fellow-travellers are known to be behind, as these light-fingered gentlemen slip into their places so silently as to be frequently unperceived, nor do they come alone, as a rule, so that detection of the possessor of the purse is exceedingly difficult. Omnibus proprietors owe it to the public to establish a system of detective inspectors, who by irregular visits to all vehicles would speedily lay by the heels the hundreds of rascals who now steal with impunity on all omnibuses. Hundreds of thefts of this kind take place weekly, and unless some such system is enforced at once the public will shun omnbiuses like the plague as the only means of defence available. The evil is too serious to longer remain without a remedy.

